# Ultra-sensitive detection of kanamycin for food safety using a reduced graphene oxide-based fluorescent aptasensor

**DOI:** 10.1038/srep40305

**Published:** 2017-01-05

**Authors:** Na-Reum Ha, In-Pil Jung, Im-Joung La, Ho-Sup Jung, Moon-Young Yoon

**Affiliations:** 1 Department of Chemistry and Research Institute of Natural Sciences, Hanyang University, Seoul 04763, Republic of Korea; 2 Food Safety Center, Lotte Confectionery Co., Ltd., Seoul 07207, Republic of Korea; 3 Institute of Advanced Machinery and Design, Department of Mechanical and Aerospace Engineering, Seoul National University, Seoul 08826, Republic of Korea

## Abstract

Overuse of antibiotics has caused serious problems, such as appearance of super bacteria, whose accumulation in the human body through the food chain is a concern. Kanamycin is a common antibiotic used to treat diverse infections; however, residual kanamycin can cause many side effects in humans. Thus, development of an ultra-sensitive, precise, and simple detection system for residual kanamycin in food products is urgently needed for food safety. In this study, we identified kanamycin-binding aptamers via a new screening method, and truncated variants were analyzed for optimization of the minimal sequence required for target binding. We found various aptamers with high binding affinity from 34.7 to 669 nanomolar *K*_*d*_^*app*^ values with good specificity against kanamycin. Furthermore, we developed a reduced graphene oxide (RGO)-based fluorescent aptasensor for kanamycin detection. In this system, kanamycin was detected at a concentration as low as 1 pM (582.6 fg/mL). In addition, this method could detect kanamycin accurately in kanamycin-spiked blood serum and milk samples. Consequently, this simple, rapid, and sensitive kanamycin detection system with newly structural and functional analysis aptamer exhibits outstanding detection compared to previous methods and provides a new possibility for point of care testing and food safety.

Antibiotics have been widely used in animals to cure various diseases and as growth promoters^1^. However, antibiotics can accumulate in the human body through the food chain, which may cause a variety of side effects^1^ and has resulted in the appearance of “super bacteria” with antibiotic tolerance^2^. Therefore, it is important to analyze the maximum residue limits (MRLs) of residual antibiotics in foodstuffs.

Kanamycin, an aminoglycoside antibiotic, is widely used in veterinary medicine for the treatment and prevention of microbial infections^3^. Although kanamycin plays an effective role in bacterial treatment, it can cause many side effects, such as loss of hearing, kidney toxicity, allergic reactions, and antibiotic resistance in humans^4^. Therefore, a sensitive and selective technique for detection of kanamycin in animal body fluids and foods (e.g. milk) is required. The Korea Food and Drug Administration (KFDA) fixed the maximum residue limit (MRL) of kanamycin in milk as 0.1 ppm (0.1 mg/kg), and the European Union (EU) has established an MRL of 0.15 ppm (0.15 mg/kg) for milk^3^,^5^. The residual amount of kanamycin in foodstuff can lead to antibiotic resistance from pathogenic bacterial strains, which can endanger the consumer^6^. For the purpose of observing residual kanamycin levels in animal-derived food products, simple and trustworthy analytical methods are required. Thus, it is necessary to develop a highly sensitive and selective diagnostic system for residual kanamycin in food or other products. At present, various analytical methods for kanamycin detection have been used, such as high-performance liquid chromatography (HPLC)^7^, gas chromatography-mass spectrometry (GC-MS)^8^, surface plasmon resonance (SPR)^9^, capillary electrophoresis (CE)^10^, microbiological methods^11^, and ELISA^12^,^13^. Most of these methods are time-consuming and require expensive equipment and strong technical expertise^3^,^14^.

Aptamers are essentially short RNA or single-stranded DNA oligonucleotides (usually 20–80 nucleotides with 6–30 kDa molecular weights) that can bind to target molecules with high binding affinity and specificity and fold into unique three-dimensional conformations^15^,^16^. Aptamers are selected by the *in-vitro* process SELEX (systematic evolution of ligands by exponential enrichment)^17^ and bind to various targets, ranging from small molecules including antibiotics and endocrine disruptors to proteins and cells^18^,^19^. As aptamers are quite small biological molecules in comparison with antibodies, they have been widely used in biological detection systems with various advantages, including ease of production and modification, low cost, and lack of toxicity and immunogenic property^15^,^20^,^21^.

Graphene, a two-dimensional (2D) carbon crystal with a single carbon atom thickness arranged in six-membered rings, has attracted due to its remarkable electronic, mechanical, optical, and thermal properties^22^. Graphene oxide (GO) was prepared by oxidizing graphite with strong oxidants and then converting to reduced graphene oxide (RGO)^23^,^24^. Because of their unique electronic properties, GO and RGO function as strong energy acceptors based on either electron or energy transfer mechanisms^25^. Generally, RGO exhibits good water dispersibility and an outstanding fluorescence quenching effect owing to the presence of more crystalline graphene regions on the sheets compared with GO^26^,^27^. Recently, graphene and its derivatives have been used as a platform for the detection of DNA, miRNA, proteins, nucleases, small molecules, and metal ions^28^. A fluorescence-labeled ssDNA probe is adsorbed well and efficiently quenched by the GO/RGO surface, while dsDNA does not bind to GO/RGO^29^. Because of hydrophobic and π-π stacking interactions between the rings of nucleobases and the hexagonal surfaces of the GO/RGO, ssDNA can stably adsorb onto the graphene surface. However, dsDNA maintains its helical structure, so effective shielding of the nucleobases within the densely negatively-charged phosphate backbone of the rigid dsDNA or ssDNA aptamer-target complex blocks the interaction with a graphene surface^30^. These properties have been used for the detection of various targets based on differences in fluorescence quenching degree.

Herein, we identified a new kanamycin binding aptamer by SELEX and optimized its structure to the minimal size required for proper binding to a target by aptamer truncation and molecular docking study. Furthermore, we developed an ultra-sensitive, specific, simple, and rapid RGO-based fluorescent aptasensor for detection of kanamycin. Our system was also tested using various real samples like blood serum, and animal-derived food, especially milk. We suggest that our technique could be applied to a point of care system for detection of kanamycin residues and other recognition molecules.

## Results and Discussion

### Immobilization of kanamycin on solid support

Kanamycin was immobilized on NOS (N-Oxysuccinimide ester)-coated 96-well plates via reaction of the primary amine group on kanamycin with the NOS group, thereby forming an amide bond. To validate the fixation of kanamycin on the plate, we first synthesized the signal detection DNA probe (5′Biotin – TTTTTT-C_6_-NH_2_3′). Because the thymine nucleotide did not have a primary amine, the signal detection DNA probe could bind the NOS-coated 96-well plate with directional bias. Thus, signal detection DNA probe with amine group modified reacted the NOS group, and the streptavidin conjugated horseradish peroxidase (HRP) was treated for signal developing process with 3,3′,5,5′-tetramethyl benzidine (TMB). As shown in Fig. S1A, the optimum concentration of signal detection DNA probes was 25 nM.

To determine the proper concentration of kanamycin, various concentrations of kanamycin were immobilized on an NOS-coated 96-well plate, and then 25 nM of signal detection DNA probe was applied. As the concentration of kanamycin increased, the absorbance decreased because the binding position of the signal detection DNA probe was diminished. The optimal concentration of kanamycin required for fixation on the NOS-coated plate was 0.5 mM (Fig. S1B). This suggests that the kanamycin was sufficiently immobilized on the NOS-coated plate, and the fixation concentrations of the other antibiotics were also tested.

### Identification of kanamycin binding aptamer (KBA) and structural analysis

**Figure 1:**
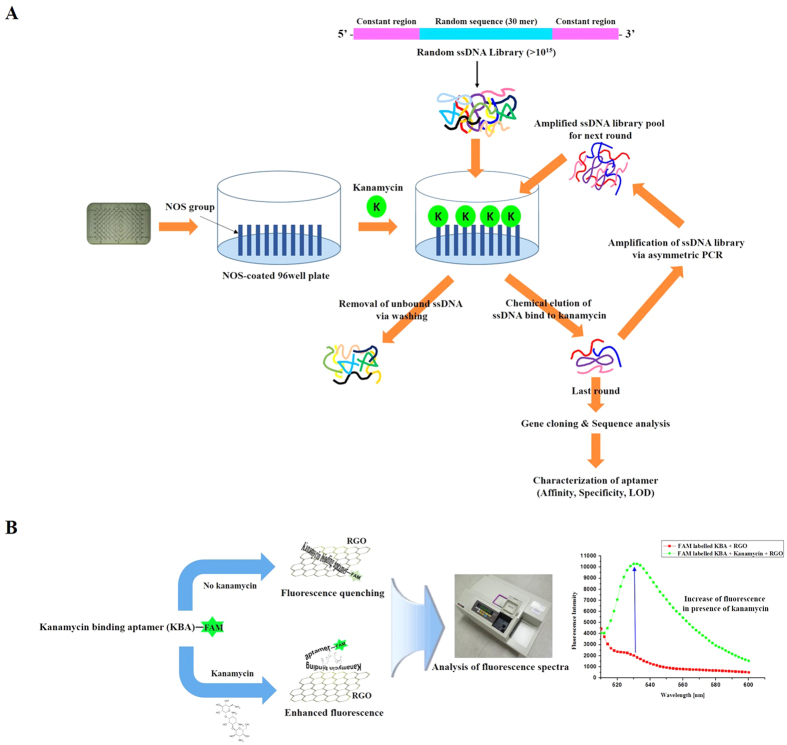
Overall schematic of (**A**) the systematic evolution of ligands by exponential enrichment (SELEX) method for the selection of aptamers against kanamycin, and (**B**) reduced graphene oxide (RGO)-based fluorescent aptasensor for kanamycin detection.

Kanamycin on an NOS-coated plate and the ssDNA library obtained by asymmetric PCR were used for aptamer screening, and nine rounds of the SELEX process were performed (Fig. 1A, Table S1). After completing the SELEX process, three kanamycin binding aptamers (KBA 1, KBA 2, and KBA 3) with different random 30-base sequences were found (Table S2).

**Figure 2:**
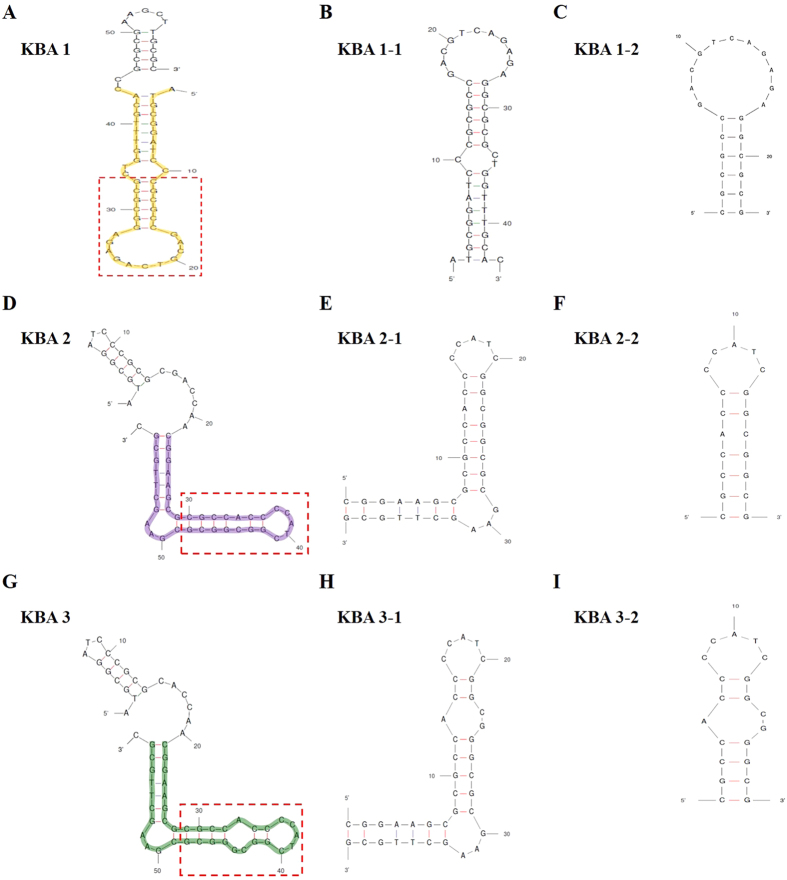
Schematic representation of the secondary structures of selected kanamycin binding aptamers (KBAs) and its truncation form generated by the M-fold program. (**A–C**) Derivatives of KBA 1, (**D–F**) derivatives of KBA 2, and (**G–I**) derivatives of KBA 3. 5′ or 3′ primer binding sites and several unpaired terminal nucleotides were eliminated, and the highlighted region indicates the first truncation form (**B,E** and **H**). Additional truncations of stem-loop motifs are shown in the red dot box (**C,F** and **I**).

To identify the high-binding affinity domains in the post-screened aptamers, the structure of each aptamer was analyzed, and truncation of the stem-loop regions was conducted (Fig. 2, Table S2,). Each original aptamers displayed a complex hairpin stem-loop secondary structure with several unpaired terminal nucleotides. To identify the importance of stem-loop regions in kanamycin binding, the primer binding region on folded structure and unpaired nucleotide on the terminal end was truncated, and we obtained the first truncation aptamers (KBA 1-1, 2-1, and 3-1). Further truncation (second truncation) to yield small hairpin loops (KBA 1-2, 2-2, and 3-2) was performed.

### Characterization of truncated aptamer (binding affinity, specificity, limit of detection)

**Figure 3:**
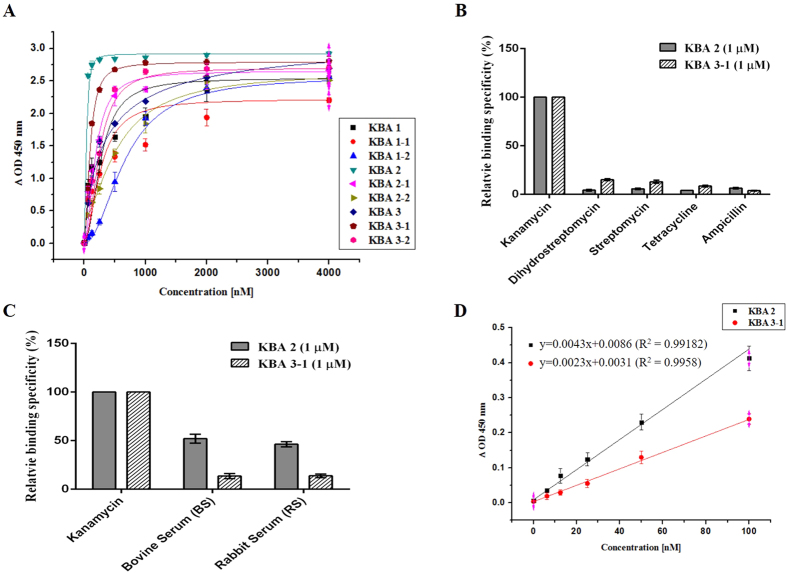
Determination of binding affinity, specificity against various antibiotics and serum, and limit of detection against kanamycin. (**A**) Binding affinities of KBAs toward kanamycin. The *K*_*d*_^*app*^ value for each KBA was determined by ELONA. (**B**) Binding specificity of KBA 2 and KBA 3-1 (1 μM), which showed lower binding affinities toward other antibiotics than the other KBAs. (**C**) Binding specificity of KBA 2 and KBA 3-1 (1 μM) against 1:10 diluted bovine serum and rabbit serum. (**D**) The LOD values of KBA 2 and KBA 3-1 were analyzed. Both aptamers could detect kanamycin down to 6.25 nM. All experiments were performed in triplicate.

The aptamer binding affinity was measured by ELONA (enzyme-linked oligonucleotide assay) using biotin-labeled KBAs. As described in Table S2 and Fig. 3A, the apparent binding affinities (*K*_*d*_^*app*^) of KBAs were calculated to be in the concentration range of 34.7 nM to 669 nM. Among the KBAs, native KBA 2 exhibited the lowest *K*_*d*_^*app*^ value (34.7 nM). KBA 1-1 showed a similar *K*_*d*_^*app*^ value to that of KBA 1, indicating that the reverse primer binding region in KBA 1 did not have any effect on kanamycin binding. Also, the other initial truncation aptamer, KBA 3-1 displayed a four-fold increase in binding affinity (92.3 nM) compared to that of KBA 3 (341 nM). This increase in binding affinity could be due to the removal of non-binding nucleotides that hinder the binding process and whose deletion may lead to a more favorable secondary conformation for kanamycin binding in the truncated aptamer. Further deletion of nucleotides (second truncation) to form a small hairpin loop (KBA 1-2, KBA 3-2) did not show any improvement in the binding affinity.

The target specificity test was performed with the same procedure used for the binding affinity test against various antibiotics (streptomycin, dihydrostreptomycin, tetracycline, and ampicillin) and Tris. As shown in Fig. 3B and Fig. S2, most of the KBAs showed very high target specificity. In particular, KBA 2, KBA 3, and KBA 3-1 exhibited remarkable target binding specificity to other antibiotics.

The specificity of each candidate aptamer was measured in complex samples of bovine serum and rabbit serum. KBA 3-1 showed very low binding ability to both sera. However, KBA 2 exhibited an approximate 50% binding property to these sera (Fig. 3C). Though KBA 2 showed high target binding specificity to various antibiotics, it showed low specificity in blood serum.

The limit of detection (LOD) for KBA 2 and KBA 3-1 was also analyzed, as these aptamers have better binding affinity compared to other KBAs and derivatives. Kanamycin was detected by both aptamers at a concentration as low as 6.25 nM (Fig. 3D). These results demonstrated that KBA 3-1 is the minimal sequence required to provide high kanamycin binding affinity and specificity.

### Binding site of kanamycin to aptamer

**Figure 4:**
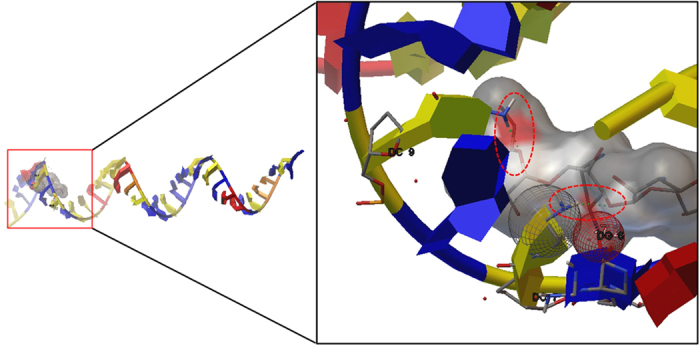
Molecular docking between KBA 3-1 and kanamycin. The hydrogen bonds between KBA 3-1 and kanamycin are indicated by red circles. The hydrophobic interaction patch is indicated by the gray sphere. The red sphere indicates ionic interaction of hydroxyl groups.

The binding site of KBA 3-1 was estimated by molecular docking analysis. The 3D-structure of KBA 3-1 was constructed as an α-helix and was used as a receptor molecule. Molecular docking analysis revealed that the ligand (kanamycin) bound to the guanidine or cytidine consisting of stem in KBA 3-1 (Fig. 4), as seen in the estimation of 2-D structure of KBA 3-1. Based on this result, truncation of the forward primer binding region on KBA 3 maintains the interaction between stem structure in KBA 3-1 and kanamycin. Thus, further detection analysis was carried out using KBA 3-1.

### Sensing principle of RGO-based fluorescent aptasensor for kanamycin detection

A biological sensing system based on the fluorescent aptasensor for kanamycin was developed using kanamycin-induced fluorescent signal changes. In the presence of kanamycin, kanamycin preferentially binds to KBA 3-1 because of the electrostatic, hydrogen bond, and spatial matching effect that together result in high affinity. The complex of kanamycin-KBA 3-1 prevents the adsorption of RGO. Figure 1B displays the strategy for detection of kanamycin using the RGO-aptamer based sensor. The 5′ FAM-labeled KBA 3-1 was adsorbed on the surface of RGO via π-π stacking interactions. Consequently, the fluorescence of FAM was efficiently quenched by RGO via fluorescence resonance energy transfer (FRET) between RGO as a quencher and FAM as a fluorophore. In the presence of kanamycin, KBA 3-1 could change its conformation upon binding to kanamycin. The kanamycin-KBA 3-1 complex induced desorption of KBA 3-1 from the surface of RGO; as a result, the fluorescence of FAM-labeled KBA 3-1 increased. Therefore, we developed a novel fluorescent aptasensor for kanamycin using a target specific aptamer (KBA 3-1) and RGO.

### Optimization of the experimental conditions

Prior to the detection of kanamycin using the RGO-based fluorescent aptasensor, various experimental factors such as RGO concentration, RGO/target incubation time, and reaction pH were analyzed. First, various concentrations of RGO (0.01–4 mg/mL) were added into a 96-well white plate containing 80 μL of 1X PBS (pH 8.5) at a fixed concentration of 1 μM of KBA 3-1 (100 nM final concentration). As shown in Fig. S3A, 0.1 mg/mL of RGO (final concentration) was used for the maximum fluorescence quenching ability, and this amount was sufficient to quench the fluorescent signal obtained by 100 nM KBA 3-1.

The optimal incubation time was determined by incubation of 0.1 mg/mL of RGO for different times (5–60 min) with KBA 3-1 and kanamycin complex. As shown in Fig. S3B, RGO reacted with the kanamycin-KBA 3-1 complex and obtained maximum fluorescence within 25 min. To determine the optimal KBA 3-1 and kanamycin incubation time, KBA 3-1 (1 μM) and kanamycin (10 μM) were incubated together for various times (1–60 min), and then 0.1 mg/mL of RGO was added for 25 min. As displayed in Fig. S3C, the maximum reaction with kanamycin and FAM-labeled KBA 3-1 occurred within 30 min.

The conformational stability of KBA 3-1 and fluorescence intensity of FAM were also affected by the reaction pH. Hence, 1X PBS with different pH values from 6.0 to 10.0 was used to evaluate the effects of pH on the reaction of KBA 3-1 (1 μM), kanamycin (10 μM), and RGO (0.1 mg/mL). As observed in Fig. S3D, the fluorescence intensity gradually increased from 6.0 to 8.0, slightly increased and plateaued at pH 8.5, and then decreased when the pH exceeded 8.5. Therefore, 1X PBS (pH 8.5) was used for further experiments.

To evaluate the effects of reaction temperature, KBA 3-1 (1 μM) and kanamycin (10 μM) were incubated for 30 min, and then 0.1 mg/mL of RGO was added for 25 min in 1X PBS (pH 8.5). The reaction was carried out at various temperature ranging from 4 to 45 °C (4, 16, 23, 30, 37, 45 °C). As shown in Fig. S3E, the maximum fluorescence signal was obtained at room temperature (23 °C), and the fluorescence intensity decreased gradually along with the increase in temperature. It seems that the reaction kinetics in aptamer-kanamycin are slow at the low temperature. Therefore, the optimum reaction temperature (23 °C) was determined.

### Sensitivity and selectivity of the detection of kanamycin using RGO-based fluorescent aptasensor

**Figure 5:**
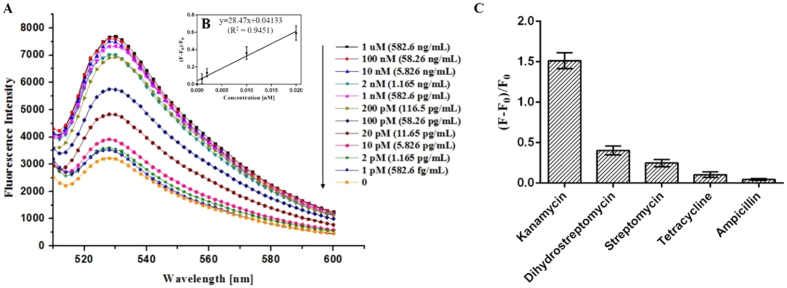
Analytical performance of the RGO-based fluorescent aptasensor for kanamycin. (**A**) Fluorescence spectra of 100 nM FAM-labeled KBA 3-1 and 0.1 mg/mL of RGO in 1X PBS (pH 8.5) containing various concentrations of kanamycin (1 pM–1 μM). (**B**) The peak fluorescence change is linear with kanamycin concentration over the range from 1 to 20 pM. (**C**) Selectivity of the RGO-based fluorescent aptasensor was measured at the same concentration using various antibiotics (1 μM).

The above optimized conditions were applied to the detection of kanamycin. A series of different kanamycin concentrations were incubated with KBA 3-1, and their fluorescence spectra were monitored. As illustrated in Fig. 5A, the fluorescence intensity was enhanced with increasing concentration of kanamycin from 0 to 1 μM. Further increases in kanamycin concentration above 1 μM showed no further fluorescence enhancement. In the linear range from 1 pM (582.6 fg/mL) to 20 pM (11.65 pg/mL) (Fig. 5B, inset), the LOD for kanamycin was calculated to be 1 pM on the basis of the 3 σ/slope. All experiments were carried out three times under identical conditions. Furthermore, the detection performance of the suggested assay was compared with those of other reported methods. The RGO-based fluorescent aptasensor exhibited superior detection sensitivity (LOD) against kanamycin, as low as 1 pM, compared to other previous detection methods (Table 1).

To investigate the selectivity of the RGO-based fluorescent aptasensor, we evaluated the detection of other antibiotics under the same experimental conditions used for kanamycin. As shown in Fig. 5C, this aptasensor system showed a very slight fluorescence increase in the presence of other antibiotics. More specifically, this RGO-based fluorescent aptasensor exhibited negligible fluorescence in the presence of ampicillin. The outstanding selectivity of the aptasensor was a virtue of the high specificity of KBA 3-1. This indicated that the RGO-based fluorescent aptasensor responded selectively to kanamycin over other tested antibiotics.

### Detection of kanamycin in real samples by the RGO-based fluorescent aptasensor

**Figure 6:**
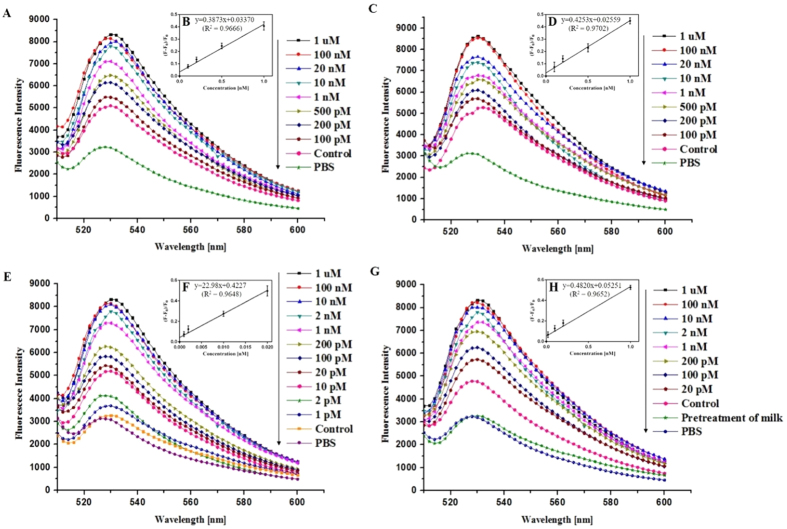
Detection of kanamycin in various real samples using the RGO-based fluorescent aptasensor system. Fluorescence spectra of 100 nM FAM-labeled KBA 3-1 and 0.1 mg/mL of RGO in (**A**) bovine serum (BS) and (**C**) rabbit serum (RS) containing various concentrations of kanamycin (100 pM–1 μM). (**B,D**) Peak fluorescence change shows a linear relationship with kanamycin concentration from 100 pM to 1 nM. (**E**) Fluorescence spectrum of 100 nM FAM-labeled KBA 3-1 and 0.1 mg/mL of RGO with pretreatment of milk containing various concentrations of kanamycin (1 pM–1 μM). (**F**) Peak fluorescence change shows a linear relationship with kanamycin concentration from 1 to 20 pM. (**G**) Fluorescence spectrum of 100 nM FAM-labeled KBA 3-1 and 0.1 mg/mL of RGO in real milk containing various concentrations of kanamycin (20 pM–1 μM). (**F**) Peak fluorescence change shows a linear relationship with kanamycin concentration from 20 pM to 1 nM.

The possible applicability of the sensor in the detection of kanamycin in real samples was investigated by analysis of artificially contaminated sample such as kanamycin-spiked blood sera (bovine and rabbit), pre-treated milk, and actual milk samples. The experiments were carried out by adding a series of known quantities of kanamycin into each sample and then using the developed detection method to evaluate the applicability and analytical validity of the sensor system. As shown in Fig. 6A and C, this RGO-based fluorescent aptasensor system could detect kanamycin in kanamycin-spiked both sera (bovine and rabbit) down to 100 pM. Although the detection sensitivity in both blood sera was about 100-fold higher than that of standard kanamycin samples, this method showed accurate detection owing to the high specificity of KBA 3-1.

To compare the detection performance ability of an aptamer toward kanamycin in the proposed sensor system, KBA 2 was applied to the RGO-based fluorescent aptasensor. The aptasensor using KBA 2 exhibited similar detection to the KBA 3-1 aptasensor system (Fig. S4). More specifically, the detection sensitivity was as low as 100 fM, and this value was 10-fold greater than the KBA 3-1-based aptasensor (Fig. S4A). Also, the detection specificity against various antibiotics showed a similar pattern to the KBA 3-1 system (Figs S4C and 5C). This result corresponded well with the KBA 2 properties (Fig. 3B). However, as shown in Fig. S5, the aptasensor based on KBA 2 showed a relatively high background signal in both blood sera compared to the aptasensor based on KBA 3-1. Thus, the detection sensitivity (LOD) of the aptasensor based on KBA 2 was about 5 nM. This result is due to the low target specificity of KBA 2 in blood serum. As shown in Fig. 3C, KBA 2 showed low specificity within bovine and rabbit serum. Thus, the high LOD value of the KBA 2-based fluorescent aptasensor corresponded well with the previous result of low KBA 2 specificity.

In addition, to evaluate this RGO-based fluorescent aptasensor applicability to other real samples, the described method was applied for the analysis of kanamycin-spiked in raw milk. To examine the detection effects within milk, the sensing system was divided into two parts: i) pretreated milk and ii) raw milk.

Kanamycin-spiked pretreated milk samples (Pretreated milk samples containing different concentrations of kanamycin) were tested. As shown in Fig. 6E, the fluorescence signals gradually increased with increasing kanamycin concentration within the pre-treated milk samples. This general tendency was the same for the kanamycin standard solutions, and a 1 pM detection limit was obtained in the pre-treated milk samples. This indicates that the milk components after pretreatment did not interfere with kanamycin detection. Furthermore, the sensing of kanamycin-spiked in raw milk was also carried out. The fluorescence spectrum without kanamycin increased due to the non-specific binding to the milk matrix (Fig. 6G). Nevertheless, the kanamycin in spiked raw milk could be detected down to 20 pM. Moreover, the detection sensitivity was much lower than the maximum residue level (MRL) of kanamycin in milk (0.1 ppm, about 171 nM) established by KFDA.

In addition, the recovery of kanamycin in spiked real samples (bovine serum, rabbit serum, and raw milk) and the relative standard deviation (RSD) (n = 3) are presented in Table 2. These results clearly show that our RGO-based fluorescent aptasensor is sufficient to detect kanamycin residue in complex, real samples.

Even though the complex components are existed and certain biological molecules in blood serum or milk could be adsorbed on the RGO surface, it is not an obstacle for adsorbing of FAM-labeled ssDNA and diminishing of fluorescence quenching. Through the application of kanamycin detection in bovine and rabbit sera, the RGO-based fluorescent aptasensor could be further used as a rapid, point of care kanamycin detection system using pre-treated blood at the time of animal slaughter. In addition, this system might be useful to evaluate food freshness. Therefore, this suggested method could be a promising technique for food safety analysis and prevention of overuse of antibiotics or other harmful reagents in the medical field.

## Conclusion

In summary, we successfully developed a novel method for the detection of kanamycin using a reduced graphene oxide-based fluorescent aptasensor system. In this paper, we identified and evaluated potent ssDNA aptamers as a detection tool for residual kanamycin. Among these aptamers, KBA 3-1 showed a 92.3 nM *K*_*d*_^*app*^ value, target specificity, and a low detection limit of 6.25 nM. Furthermore, the RGO-KBA 3-1-based fluorescent aptasensor was constructed to detect kanamycin. The proposed system could accurately detect kanamycin in standard, blood serum, and real milk samples with detection limits of 1 pM, 100 pM, and 20 pM respectively. In addition, all experimental processes were completed within 1 h. This demonstrates that the designed system allows for the rapid detection of kanamycin. These results indicate that this method is an ultra-sensitive detection strategy for kanamycin in real samples such as foodstuffs for food safety and sanitation.

## Methods

### Immobilization of kanamycin on a solid support

*In-vitro* aptamer selection for kanamycin was carried out on an NOS (N-oxysuccinimide ester)-coated DNA-Bind 96-well plate. Then, 0.5 mM of kanamycin dissolved in 1X PBS (pH 8.5) was immobilized on an NOS-coated 96-well plate for 2 h via formation of amide bonds between primary amines in kanamycin and NOS groups. The wells were washed three times with 1X PBST (PBS containing 0.1% Tween-20), and the unreacted NOS groups on the plate were then incubated with blocking buffer (1 M Tris, pH 8.5) at room temperature for 1 h to block non-specific interactions. After five gentle washes, the aptamer screening platform for kanamycin was prepared. For negative selection, an NOS-coated 96-well plate with tetracycline was also prepared.

### Aptamer screening for kanamycin

Total 60 mer of synthetic single-stranded DNA (ssDNA) library containing 30 bases of random nucleotide was amplified by asymmetric PCR as described previously^15^. The preprared ssDNA library (500 pmole) dissolved in 1 mL of binding buffer (20 mM Tris-HCl, 5 mM KCl, 5 mM MgCl_2_, 50 mM NaCl, pH 8.0) was denatured for 15 min at 95 °C and then immediately cooled to 4 °C for 10 min. Thereafter, the ssDNA library was added to a kanamycin-coated 96-well plate (50 pmole/well), and incubation was carried out for 90 min in the first round. The unbound ssDNA library was removed through seven washing steps using 200 μL of binding buffer each time. The bound ssDNA library was eluted with 200 μL of 20 mM NaOH for 20 min. Then the eluted ssDNA was neutralized with 20 mM HCl and precipitated by ethanol. After precipitation, the ssDNA library was amplified by asymmetric PCR. The amplified ssDNA library was used as a DNA pool for the next selection round. In order to increase the specificity of the ssDNA aptamer, negative selection was performed with Tris and tetracycline during rounds 6 and 7 of SELEX, and then the unbound ssDNA was collected and precipitated. A total of nine rounds of SELEX were performed under gradually harsher conditions (Table S1).

### Sequence and structural analysis of aptamer

The ssDNA library pool obtained from the final round of SELEX was amplified by symmetric PCR and then inserted into the pET28a (+) vector. The sequence analysis of ligation product was determined from Macrogen (Seoul, Korea). The secondary structures of the ssDNA aptamers and its truncation forms were predicted using M-fold free software^36^.

### Determination of binding affinity, specificity, and limit of detection

The apparent binding affinities of KBAs were measured by ELONA with 5′-biotin-conjugated KBAs (biotin-KBAs) synthesized by Bioneer and streptavidin-conjugated horseradish peroxidase (HRP) antibody (BD Biosciences, Franklin Lakes, NJ, USA). 0.5 mM of kanamycin, dissolved in 1X PBS (pH 8.5), was immobilized on an NOS-coated 96-well plate for 2 h. The wells were washed three times with 1X PBST (pH 8.5) and then incubated with 1 M Tris for 1 h. After five gentle washes, each biotin-KBA was added in a concentration-dependent manner, and incubation was carried out for 2 h. The unbound aptamers were removed through 10 washing steps. Then, streptavidin-conjugated HRP (1:2000 dilution in 1X PBST) was added for 1 h, and bound KBAs were detected by addition of 3,3′,5,5′-tetramethyl benzidine (TMB) solution (R&D Systems, Minneapolis, MN, USA). After 15 min incubation, the reaction was terminated by adding 1 M H_2_SO_4_, and the absorbance was measured using a SpectraMax M2 microplate reader (Molecular Devices, Sunnyvale, CA, USA) at 450 nm. The binding affinities for KBAs were estimated based on the apparent binding ability to kanamycin and Tris using the Origin Pro program.

The binding specificity of KBAs toward kanamycin was also evaluated by measurement of binding ability to other antibiotics, such as streptomycin and dihydrostreptomycin as aminoglycoside antibiotics, as well as tetracycline and ampicillin as a different class of antibiotics. The limit of detection (LOD), as described by the International Union of Pure and Applied Chemistry (IUPAC), was calculated with Equation (1).


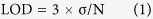


In the equation, σ is the standard deviation of a blank, and N is the slope of the fitted line.

### Molecular docking of aptamer

The 3D-structures of aptamers were estimated and constructed by the Discovery Studio 3.5 Visualizer from BioVia (San Diego, CA, USA). The 3D-structures of aptamers were established as α-helical single-stranded DNA and adjusted by Molecular Dynamics with Abalone software (Stockholm, Sweden). The aptamer was corrected with the background of water, and the flip angle of the aptamer was restricted up to 15°.

Molecular docking analysis of kanamycin against aptamers was performed by Auto Dock Vina^37^. We selected an estimated 3D-structure of each aptamer as a receptor protein. The flexible and non-flexible residues were identified, and the appropriate charges were added. The ligands were modeled using all free torsional bonds in the structure. The docking area was calculated using a three-dimensional grid box with grid points of 120 × 120 × 120 Å and a line spacing of 0.375 Å.

### General procedure of fluorescent sensing of kanamycin

For kanamycin detection, 70 μL of 1X PBS (pH 8.5) was added into a 96-well white plate. FAM-labeled KBA (100 nM) was initially added to form the kanamycin-aptamer complex. A series of kanamycin concentrations in 1X PBS (pH 8.5) (from 1 pM to 1 μM) were separately added and incubated with gentle shaking for 30 min at room temperature. Then, 10 μL of RGO (0.1 mg/mL) was added to the kanamycin-KBA mixture. The solution was mixed well and shake-incubated for another 25 min at room temperature. Next, the fluorescence intensities (λ_Ex_ = 495 nm, λ_Em_ = 520 nm) of the biological sensing system were recorded using a SpectraMax M2 microplate reader to measure the effects of kanamycin concentration on fluorescence strength.

### Investigation of the sensitivity and selectivity of the aptasensor

The sensitivity of the RGO-based fluorescent aptasensor was tested with various kanamycin concentrations. The LOD was calculated with Equation (1), and the selectivity was analyzed in the presence of 1 μM (final concentration) streptomycin, dihydrostreptomycin, tetracycline, and ampicillin using the same procedure described above for kanamycin.

### Analysis of kanamycin in blood serum, and milk with the developed aptasensor

The reliability of the RGO-based fluorescent aptasensor in practical applications was measured by determining the fluorescence signal in kanamycin-spiked blood serum (bovine serum, rabbit serum), milk samples, and pre-treated milk samples. Pretreatment of milk was prepared according to the previous study^38^. Artificially contaminated serum and milk were prepared by spiking a standard kanamycin solution into the samples and then measuring the fluorescence signal. Then, the RGO-based fluorescent assay was carried out as described above.

## Additional Information

**How to cite this article**: Ha, N.-R. *et al*. Ultra-sensitive detection of kanamycin for food safety using a reduced graphene oxide-based fluorescent aptasensor. *Sci. Rep.* **7**, 40305; doi: 10.1038/srep40305 (2017).

**Publisher's note:** Springer Nature remains neutral with regard to jurisdictional claims in published maps and institutional affiliations.

## Supplementary Material

Supplementary Information

## Figures and Tables

**Table 1:** Comparison of various kanamycin detection methods.

Detection methods	Strategy	Linear range (M)	Detection limit (M)	References
ELISA	Competitive direct ELISA using monoclonal antibody	—	1.89 × 10^−9^	13
Electrochemical	Aptasensor on a MWCNT-nanoporous PtTi system	8.58 × 10^−11^–1.72 × 10^−7^	6.35 × 10^−12^	31
Electrochemical	ECL “on-off-on” switch system based on the S_2_O_8_^2−^	—	45 × 10^−12^	32
Colorimetry	Unmodified AgNPs and aptamer	8.58 × 10^−8^–1.03 × 10^−6^	4.6 × 10^−9^	33
Colorimetry	Unmodified AuNPs and ssDNA aptamer	—	25 × 10^−9^	34
Fluorescence	UCNPs graphene FRET system	1.0 × 10^−11^–3.0 × 10^−9^	9.0 × 10^−12^	35
Fluorescence	AuNPs and FAM-labeled aptamer	0.8 × 10^−9^–35 × 10^−8^	0.3 × 10^−9^	4
Fluorescence	RGO-based fluorescent aptasensor	1.0 × 10^−12^–20.0 × 10^−12^	1.0 × 10^−12^	This work

**Table 2:** Determination of kanamycin in spiked samples.

Samples	Added (pM)	Detected (pM)^a^	Recovery (%)	RSD (%, n = 3)
Bovine serum	100	99.15	99.15	2.677
	200	202.13	100.07	3.484
Rabbit serum	100	100.23	100.23	2.170
	200	198.84	99.42	2.854
Raw milk	20	20.58	102.90	1.170
	200	200.83	100.42	2.182

^a^Mean values of three experiments.

## References

[b1] Qin X., Yin Y., Yu H., Guo W. & Pei M. A novel signal amplification strategy of an electrochemical aptasensor for kanamycin, based on thionine functionalized graphene and hierarchical nanoporous PtCu. Biosens. Bioelectron. 77, 752–758 (2016). 26513281 10.1016/j.bios.2015.10.050

[b2] Xing Y. P., Liu C., Zhou X. H. & Shi H. C. Label-free detection of kanamycin based on a G-quadruplex DNA aptamer-based fluorescent intercalator displacement assay. Sci. Rep. 5, 8125–8132 (2015). 25634469 10.1038/srep08125 PMC4311242

[b3] Wang Y. . Analysis of the kanamycin in raw milk using the suspension array. J. Chem. 2013, 1–4 (2012).

[b4] Chen J. . An aptamer-based signal-on bio-assay for sensitive and selective detection of kanamycin A by using gold nanoparticles. Talanta. 139, 226–232 (2015). 25882430 10.1016/j.talanta.2015.02.036

[b5] Lim C. M., Cho B. H., Chung G. S. & Son S. W. Determination of aminoglycosidies in milk by liquid chromatography with tandem mass spectrometry. Kor. J. Vet. Publ. Hlth. 36, 121–130 (2012).

[b6] Xu W. . A novel sandich-type electrochemical aptasensor for sensitive detection of kanamycin based on GR-PANI and PAMAM-Au nanocomposites. New J. Chem. 38, 4931–4937 (2014).

[b7] Blanchaert B., Jorge E. P., Jankovics P., Adams E. & Schepdael A. V. Assay of kanamycin A by HPLC wigh direct UV detection. Chromatographia. 76, 1505–1512 (2013).

[b8] Isoherranen N. & Soback S. Chromatographic methods for analysis of aminoglycoside antibiotics. J. AOAC. Int. 82, 1017–1045 (1999). 10513005

[b9] Wang X. . Determination of human urinary kanamycin in one step using urea-enhanced surface plasmon resonance light-scattering of gold nanoparticles. Anal. Bioanal. Chem. 395, 2397–2403 (2009). 19784833 10.1007/s00216-009-3134-9

[b10] El-Attug M. N., Adams E., Hoogmartens J. & Schepdael A. V. Capacitively coupled contactless conductivity detection as an alternative detection mode in CE for the analysis of kanamycin sulphage and its related substances. J. Sep. Sci. 34, 2448–2454 (2011). 21796785 10.1002/jssc.201100267

[b11] Althaus R., Berruga M. I., Montero A., Roca M. & Molina M. P. Evaluation of a microbiological multi-residue system on the detection of antibacterial substances in ewe milk. Anal. Chim. Acta. 632, 156–162 (2009). 19100896 10.1016/j.aca.2008.10.058

[b12] Chen Y. . Rapid enzyme linked immunosorbent assay and colloidal gold immunoassay for kanamycin and tobramycin in Swine tissues. J. Agric. Food Chem. 56, 2944–2952 (2008). 18393429 10.1021/jf703602b

[b13] Jin Y., Jang J. W., Han C. H. & Lee M. H. Development of immunoassays for the detection of kanamycin in veterinay fields. J. Vet. Sci. 7, 111–117 (2006). 16645333 10.4142/jvs.2006.7.2.111 PMC3242100

[b14] Ramezani M., Danesh N. M., Lavaee P., Abnous K. & Taghdisi S. M. A selective and sensitive fluorescent aptasensor for detection of kanamycin based on catalytic recycling activity of exonuclease III and gold nanoparticles. Sens. Actuators B Chem. 222, 1–7 (2016).

[b15] Ha N. R., Lee S. C., Hyun J. W. & Yoon M. Y. Development of inhibitory ssDNA aptamers for the FtsZ cell division protein from citrus cnaker phytopathogen. Process Biochem. 51, 24–33 (2016).

[b16] Sun H. & Zu Y. A highlight of recent advances in aptamer technolgoy and its application. Molecules. 20, 11959–11980 (2015). 26133761 10.3390/molecules200711959 PMC6331864

[b17] Chen D., Yao D., Xie C. & Liu D. Development of an aptasensor for electrochemical detection of tetracycline. Food Control. 42, 109–115 (2014).

[b18] Huang K. J., Liu Y. J., Shi G. W., Yang X. R. & Liu Y. M. Label-free aptamer sensor for 17β-estradiol based on vanadium disulfide nanoflowers and Au nanoparticles. Sens. Actuators B Chem. 201, 579–585 (2014).

[b19] Zhao F. . RNA aptamer based electrochemical biosensor for sensitive and selective detection of cAMP. Biosens. Bioelectron. 66, 238–243 (2015). 25437358 10.1016/j.bios.2014.11.024

[b20] Lian Y., He F., Wang H. & Tong F. A new aptamer/graphene interdigitated gold electrode piezoelectric sensor for rapid and specific detection of *Staphylococcus aureus*. Biosens. Bioelectron. 65, 314–319 (2015). 25461175 10.1016/j.bios.2014.10.017

[b21] Shan W. . An aptamer-based quartz crystal microbalance biosensor for sensitive and selective detection of leukemia cells using silver-enhanced gold nanoparticel label. Talanta. 126, 130–135 (2014). 24881543 10.1016/j.talanta.2014.03.056

[b22] Rao C. N. R., Sood A. K., Subrahmanyam K. S. & Govindaraj A. Graphene: The new two-dimensional nanomaterial. Angew. Chem. Int. Ed. 48, 7752–7777 (2009). 10.1002/anie.200901678 19784976

[b23] Zhu B. Y. . Graphene and graphene oxide: Synthesis, properties, and applications. Adv. Mater. 22, 3906–3924 (2010). 20706983 10.1002/adma.201001068

[b24] Chua C. K. & Pumera M. Chemical reduction of graphene oxide: a synthetic chemistry viewpoint. Chem. Soc. Rev. 43, 291–312 (2014). 24121318 10.1039/c3cs60303b

[b25] Swathi R. S. & Sebastian K. L. Long range resonance energy transfer from a dye molecule to graphene has (distance)-4 dependence. J. Chem. Phys. 130, 086101 (2009). 19256631 10.1063/1.3077292

[b26] Zhao H. . Fluorescent assay for oxytetracycline based on a long-chain aptamer assembled onto reduced graphene oxide. Microchim. Acta. 180, 829–835 (2013).

[b27] Loh K. P., Bao Q., Eda G. & Chhowalla M. Graphene oxide as a chemically tunable plafrom for optical applications. Nat. Chem. 2, 1015–1024 (2010). 21107364 10.1038/nchem.907

[b28] Morales-Narváez E. & Merkoçi A. Graphene oxide as an optical biosensing platform. Adv. Mater. 24, 3298–3308 (2012). 22628274 10.1002/adma.201200373

[b29] Gao Li. . Graphene oxide-DNA based sensors. Biosens. Bioelectron. 60, 22–29 (2014). 24768760 10.1016/j.bios.2014.03.039

[b30] Cui L. . Graphene oxide protected nucleic acid probes for bioanalysis and biomedicine. Chem. Eur. J. 19, 10442–10451 (2013). 23839798 10.1002/chem.201301292

[b31] Guo W., Sun N., Qin X., Pei M. & Wang L. A novel electrochemical aptasensor for ultrasensitive detection of kanamycin based on MWCNTs-HMIMPF_6_ and nanoporous PtTi alloy. Biosens. Bioelectron. 74, 691–697 (2015). 26208174 10.1016/j.bios.2015.06.081

[b32] Zhao M., Zhuo Y., Chai Y. Q. & Yuan R. Au nanoparticles decorated C_60_ nanoparticle-based label-free electrochemiluminescence aptasensor via a novel “on-off-on” switch system. Biomaterials. 52, 476–483 (2015). 25818453 10.1016/j.biomaterials.2015.02.058

[b33] Xu Y. . Colorimetric detection of kanamycin based on analyte-protected silver nanoparticles and aptamer-selective sensing mechanism. Anal. Chim. Acta. 891, 298–303 (2015). 26388390 10.1016/j.aca.2015.08.013

[b34] Song K. M. . Gold nanoparticle-based colorimetric detection of kanamycin using a DNA aptamer. Anal. Biochem. 415, 175–181 (2011). 21530479 10.1016/j.ab.2011.04.007

[b35] Li H., Sun D. E., Liu Y. & Liu Z. An ultrasensitive homogeneous aptasensor for kanamycin based on upconversion fluorescence resonance energy transfer. Biosens. Bioelectron. 55, 149–156 (2014). 24373954 10.1016/j.bios.2013.11.079

[b36] Zuker M. Mfold web server for nucleic acid folding and hybridization prediction. Nucleic Acids Res. 31, 3406–3415 (2003). 12824337 10.1093/nar/gkg595 PMC169194

[b37] Trott O. & Olson A. J. AutoDock Vina: improving the speed and accuracy of docking wigh a new scoring function, efficient optimization and multithreading. J. Comput. Chem. 31, 455–461 (2010). 19499576 10.1002/jcc.21334 PMC3041641

[b38] Zhou N., Zhang J. & Tian Y. Aptamer-based spectrophotometric detection of kanamycin in milk. Anal. Methods. 6, 1569–1574 (2014).

