# Supplementary material for: Ultra-sensitive detection of kanamycin for food safety using a reduced graphene oxide-based fluorescent aptasensor
Source: Sci Rep. 2017 Jan 5;7:40305. doi: 10.1038/srep40305 (PMC5215691; doi:10.1038/srep40305)
Supplement: Supplementary Information [file srep40305-s1.pdf]

Supplementary Information

Ultra-sensitive detection of kanamycin for food safety using a reduced graphene oxide-based fluorescent aptasensor

Na-Reum Ha[a], In-Pil Jung[a], Im-Joung La[b], Ho-Sup Jung[c], Moon-Young Yoon[a, *]

[a] *Department of Chemistry and Research Institute of Natural Sciences, Hanyang University, Seoul 04763, Republic of Korea*

[b] *Food Safety Center, Lotte Confectionery Co., Ltd., Seoul 07207, Republic of Korea*

[c] *Institute of Advanced Machinery and Design, Department of Mechanical and Aerospace Engineering, Seoul National University, Seoul 08826, Republic of Korea*

* Corresponding author:

Tel.: +82-2-2220-0946

Fax: +82-2-2298-0319

Address: *Department of Chemistry and Research institute of Natural Sciences, Hanyang University, 222 Wangsimni-ro, Seongdong-gu, Seoul 04763, Republic of Korea*

E-mail address: myyoon@hanyang.ac.kr (M.Y. Yoon).

Supplementary Figures

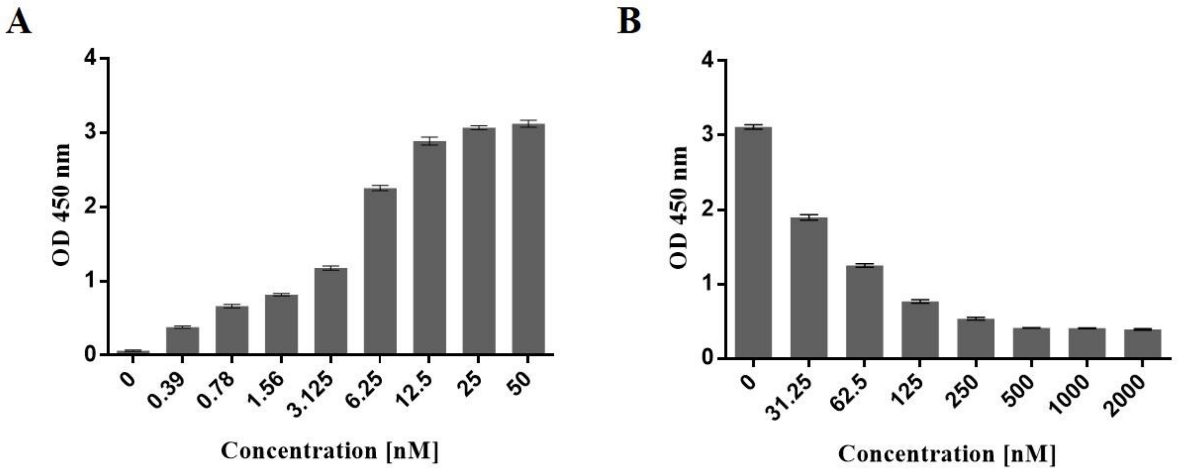

Figure S1. Determination of saturation concentrations for the signal detection DNA probe and kanamycin for DNA aptamer screening. (A) Optimization of the saturation point of the signal detection DNA probe (5’Biotin – TTTTTT-C_6-$NH_2$3’). (B) Immobilization of kanamycin on an NOS-coated DNA-BIND 96-well plate.

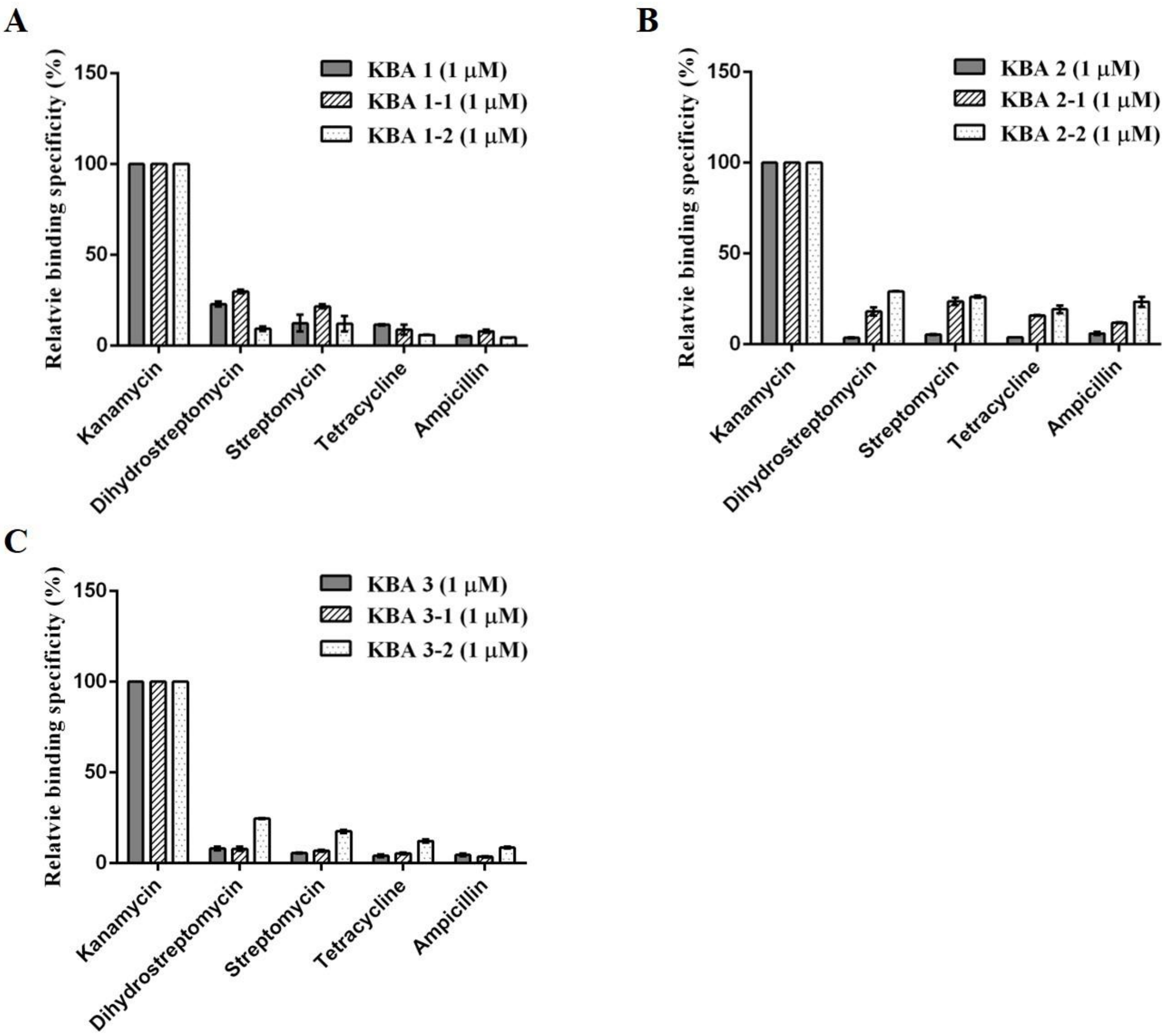

Figure S2. Antibiotic binding specificity of KBA derivatives: (A) KBA 1, (B) KBA 2, and (C) KBA 3 (1 μM, respectively).

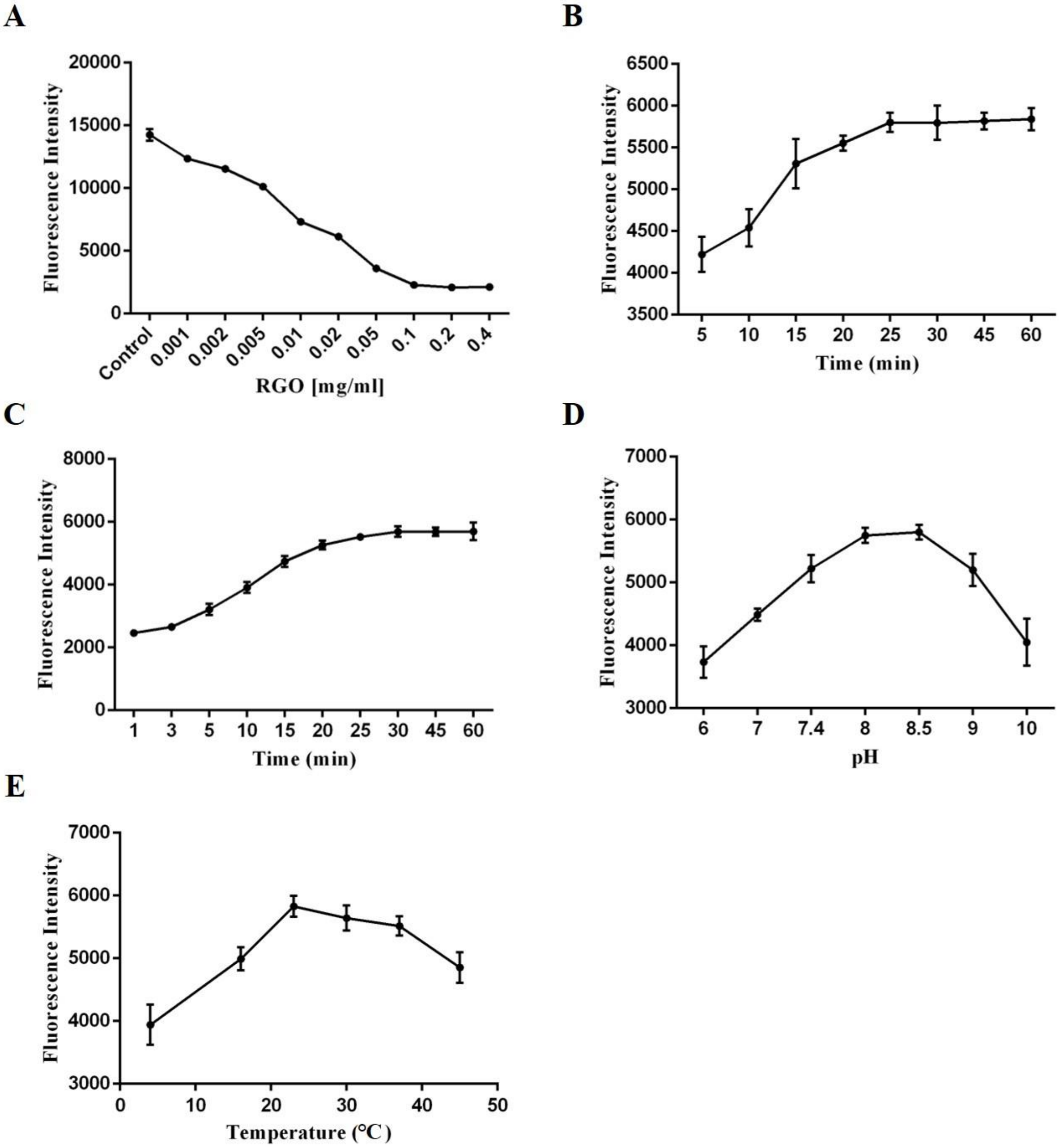

Figure S3. Optimization of the experimental conditions for the RGO-based fluorescent aptasensor for kanamycin. (A) RGO concentration, (B) incubation time of RGO, (C) reaction time of kanamycin and KBA, (D) reaction pH, and (E) reaction temperature.

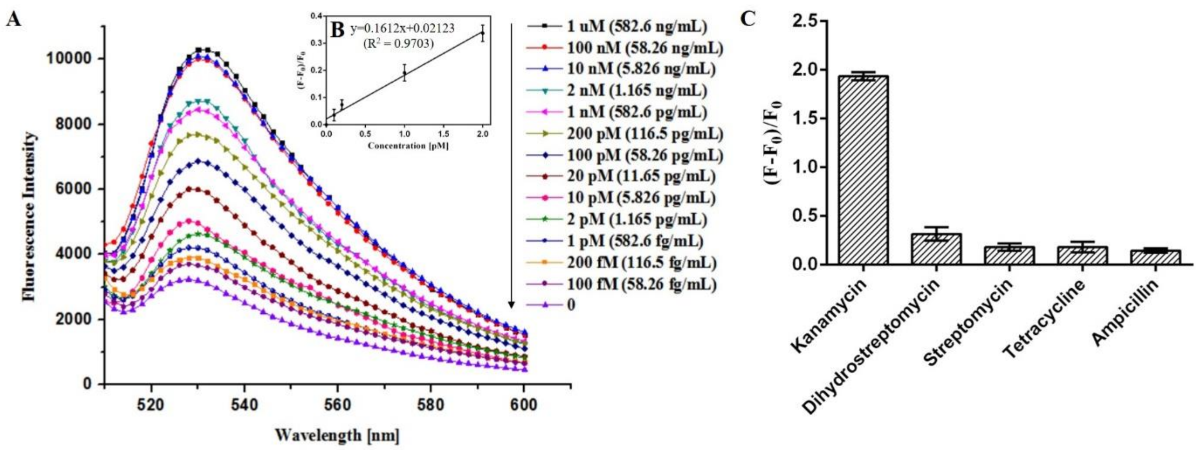

Figure S4. Analytical performance of the RGO-based fluorescent aptasensor for kanamycin using KBA 2. (A) Fluorescence spectra in the presence of 100 nM of FAM-labeled KBA 2 and 0.1 mg/mL of RGO in 1X PBS (pH 8.5) containing various concentrations of kanamycin (100 fM – 1 μM). (B) Peak fluorescence change is linear with kanamycin concentration over the range from 0.1 to 2 pM. (C) Selectivity of the RGO-based fluorescent aptasensor was measured at the same concentration of various antibiotics (1 μM).

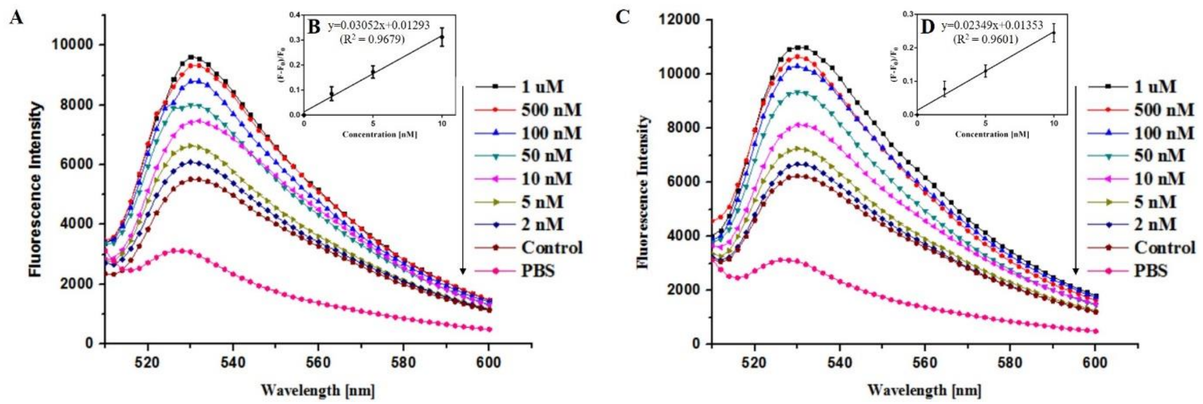

Figure S5. Detection of kanamycin in blood serum samples (bovine and rabbit serum) using the RGO-based fluorescent aptasensor system using KBA 2. Fluorescence spectra in the presence of 100 nM FAM-labeled KBA 2 and 0.1 mg/mL of RGO in (A) bovine serum (BS) and (C) rabbit serum (RS) containing various concentrations of kanamycin (2 nM – 1 μM). (B, D) Peak fluorescence change is linear with kanamycin concentration over the range from 2 to 10 nM.

Supplementary Tables

Table S1

SELEX conditions used in each round.

<table>
<tr><th rowspan="2">Round</th><th colspan="4">Buffers</th><th rowspan="2">Target [mM]</th><th rowspan="2">ssDNA (pmole/well)</th><th rowspan="2">Incubation (min)</th></tr>
<tr><th>Tris [mM]</th><th>KCl [mM]</th><th>$MgCl_2$ [mM]</th><th>NaCl [mM]</th></tr>
<tr><td>1st</td><td rowspan="9">20 (pH 8.0)</td><td rowspan="9">5</td><td rowspan="9">5</td><td>50</td><td rowspan="5">Kanamycin 0.5</td><td rowspan="9">50</td><td rowspan="3">90</td></tr>
<tr><td>2nd</td><td>100</td></tr>
<tr><td>3rd</td><td>150</td></tr>
<tr><td>4th</td><td>200</td><td rowspan="4">60</td></tr>
<tr><td>5th</td><td rowspan="3">250</td></tr>
<tr><td>6th</td><td>Tris 1000</td></tr>
<tr><td>7th</td><td>Tetracycline 10</td></tr>
<tr><td>8th</td><td>300</td><td rowspan="2">Kanamycin 0.5</td><td>45</td></tr>
<tr><td>9th</td><td>400</td><td>30</td></tr>
</table>

Table S2

Sequence information of KBAs, derivatives, and their binding affinities.

**No.**	**Aptamer sequences**[a]	**Binding affinity**[b] **(K_d^{app}, nM)**
KBA 1	ATGCGGATCCCGCGC**CGACGTCAGAGAGGC GCGCTGGTTTGCACC** GCGCGAAGCTTGCGC	268 ± 20.8
KBA 1-1	ATGCGGATCCCGCGC**CGACGTCAGAGAGGC GCGCTGGTTTGCAC**	268 ± 26.7
KBA 1-2	CGCGC**CGACGTCAGAGAGGCGCG**	669 ± 10.7
KBA 2	ATGCGGATCCCGCGC**GACCAACGGAAGCGC GCCACCCCATCGGCG**GCGCGAAGCTTGCGC	34.7 ± 35.6
KBA 2-1	**CGGAAGCGCGCCACCCCATCGGCG**GCGCG AAGCTTGCG	182 ± 57.2
KBA 2-2	**CGCCACCCCATCGGCG**GCG	564 ± 92.5
KBA 3	ATGCGGATCCCGCGC**ACCAACGGAAGCGCG CCACCCCATCGGCGG**GCGCGAAGCTTGCGC	341 ± 12.8
KBA 3-1	**CGGAAGCGCGCCACCCCATCGGCGG**GCGC GAAGCTTGCG	92.3 ± 29.1
KBA 3-2	**CGCCACCCCATCGGCGG**GCG	239 ± 52.5

[a]Total 60mer ssDNA aptamer including 30mer random nucleotides were selected, and their sequences were determined. The bold sequence indicates the random nucleotides.

[b]Binding affinities were determined as described in the Materials and Methods. The dissociation constant (K_d^{app}) was obtained from binding saturation curve fitting from three independent experiments using the Origin Pro 8 program.
